# Pre-hospital diagnosis for stroke and trauma patients using microwave technology

**DOI:** 10.1186/1757-7241-23-S2-A26

**Published:** 2015-09-11

**Authors:** Stefan Candefjord, Mikael Persson, Andreas Fhager, Bengt Arne Sjöqvist, Jan-Erik Karlsson, Mikael Elam

**Affiliations:** 1 Chalmers University of Technology, Göteborg, Sweden; 2 MedTech West, Göteborg, Sweden; 3 Sahlgrenska University Hospital, Göteborg, Sweden

## Background

For stroke and traumatic brain injury (TBI) patients minimizing the time from stroke onset/accident to treatment is fundamental to increase the chances of achieving good clinical outcome. For patients with ischemic stroke thrombolytic treatment may be effective, but only 1–8% receive this treatment due to delays in seeking medical attention and late diagnosis. TBI patients with severe injury require immediate transportation to a trauma center. Microwave technology (MWT) has potential to be used for prehospital diagnosis of stroke and TBI patients by detecting intracranial bleedings and thereby make prehospital thrombolysis for stroke patients possible and increase triage accuracy for TBI patients.

## Methods

Two clinical trials enrolling 20 + 25 stroke patients performed with research prototype systems (Brain Alfa and Stroke finderR10, Medfield Diagnostics AB, Göteborg, Sweden) have been completed. Two further studies are ongoing.

Regarding TBI laboratory experiments using a human cranium phantom and numerical simulations of subdural hematoma (SDH) have been performed. The first clinical study assessing the potential for MWT to detect SDH has recently started.

The microwave-based systems use 8–12 transmitting and receiving antennas. The classification algorithm is trained on measurements on patients with confirmed diagnosis, using a leave-one-out procedure.

## Results

For the clinical studies on stroke patients all cases of hemorrhagic stroke could be detected while correctly classifying most cases of ischemic stroke [1]. For the SDH models the total classification accuracy was 98–100%, and SDH of different sizes and at different positions could be distinguished.

## Conclusions

MWT has potential to improve the acute care for stroke and trauma patients by making a prehospital diagnosis. This would lead to decreased human suffering and large societal economic savings.
